# Anthropogenic debris in seafood: Plastic debris and fibers from textiles in fish and bivalves sold for human consumption

**DOI:** 10.1038/srep14340

**Published:** 2015-09-24

**Authors:** Chelsea M. Rochman, Akbar Tahir, Susan L. Williams, Dolores V. Baxa, Rosalyn Lam, Jeffrey T. Miller, Foo-Ching Teh, Shinta Werorilangi, Swee J. Teh

**Affiliations:** 1 Aquatic Health Program, School of Veterinary Medicine, University of California, Davis, Davis, CA 95616, USA; 2 Department of Marine Science, Faculty Marine and Fisheries Sciences, University of Hasanuddin, Makassar 90245, Indonesia; 3 Bodega Marine Laboratory and Department of Evolution and Ecology, University of California at Davis, Bodega Bay, CA 94923, USA; 4 Department of Environmental Toxicology, University of California, Davis, Davis, CA 95616, USA

## Abstract

The ubiquity of anthropogenic debris in hundreds of species of wildlife and the toxicity of chemicals associated with it has begun to raise concerns regarding the presence of anthropogenic debris in seafood. We assessed the presence of anthropogenic debris in fishes and shellfish on sale for human consumption. We sampled from markets in Makassar, Indonesia, and from California, USA. All fish and shellfish were identified to species where possible. Anthropogenic debris was extracted from the digestive tracts of fish and whole shellfish using a 10% KOH solution and quantified under a dissecting microscope. In Indonesia, anthropogenic debris was found in 28% of individual fish and in 55% of all species. Similarly, in the USA, anthropogenic debris was found in 25% of individual fish and in 67% of all species. Anthropogenic debris was also found in 33% of individual shellfish sampled. All of the anthropogenic debris recovered from fish in Indonesia was plastic, whereas anthropogenic debris recovered from fish in the USA was primarily fibers. Variations in debris types likely reflect different sources and waste management strategies between countries. We report some of the first findings of plastic debris in fishes directly sold for human consumption raising concerns regarding human health.

The ubiquity of anthropogenic marine debris and the toxicity of chemicals associated with the material have begun to raise concerns regarding how the ingestion of anthropogenic debris by marine animals may impact human health^1^. These concerns have prompted a concerted effort from government and private organizations to assess the impacts of marine debris on human and environmental health, including organizations such as NCEAS (National Center for Ecological Analysis and Synthesis), UNEP (United National Environment Programme), US EPA (United States Environmental Protection Agency), GESAMP (Joint Group of Experts on the Scientific Aspects of Marine Environmental Protection) and NOAA (National Oceanic and Atmospheric Administration). Almost every report from these groups concluded further research is required to elucidate how marine debris may be affecting humans, and thus, whether inadequate waste management strategies are coming back to haunt us in our seafood.

Due to the large presence of anthropogenic marine debris in aquatic habitats^2^,^3^,^4^,^5^ and wildlife^6^,^7^,^8^, we hypothesized that anthropogenic debris would be present in marine animals sold for human consumption. Anthropogenic marine debris is seemingly found across all habitats in the ocean, including coral reefs^9^, shallow bays^10^,^11^, estuaries^12^, the open ocean^13^,^14^ and the deep sea^15^,^16^. Anthropogenic marine debris is also found in hundreds of species globally and across multiple trophic levels^7^, including in many species of fish^7^,^17^,^18^,^19^,^20^,^21^ and bivalves^22^—animals often considered seafood. For example, Choy and Drazen^17^ found anthropogenic marine debris in the gut contents of tuna, opah and swordfish collected aboard a research vessel. Moreover, Van Cauwenberghe and Janssen^22^ found anthropogenic marine debris in commercially grown mussels and in oysters purchased from the supermarket. Although these studies found anthropogenic debris in food species, only the bivalve study indicated a direct connection between the debris and food targeted for human consumption^22^.

The likely presence of anthropogenic marine debris in seafood raises several questions regarding human health. For example, anthropogenic debris can elicit a biological response through both physical and chemical mechanisms of toxicity^23^,^24^,^25^,^26^,^27^. Small anthropogenic debris has been shown to cause physical damage leading to cellular necrosis, inflammation and lacerations of tissues in the gastrointestinal (GI) tract^27^. As such, anthropogenic marine debris may cause physical harm to humans when debris is ingested via seafood (e.g., in whole sardines, mussels and oysters). Moreover, in nature, anthropogenic debris is recovered from the marine environment with a cocktail of chemicals, including chemicals accumulated from ambient water^28^,^29^ in addition to the ingredients of the debris itself^30^. Some of these chemicals can transfer from anthropogenic debris to fish upon ingestion^25^,^31^,^32^. In turn, the ingestion of marine animals that have consumed anthropogenic marine debris has the potential to increase the burden of hazardous chemicals in humans. Furthermore, if the magnitude of adverse effects to wildlife is severe (i.e., population-level declines), food security could be impacted. The first step in understanding potential impacts of anthropogenic marine debris on human health is to determine whether anthropogenic marine debris is present in fish and shellfish caught and sold for human consumption.

Although some studies have reported the presence of marine debris in wild-caught fish commonly consumed by humans, studies demonstrating the presence of marine debris in fish directly sold at fish markets for human consumption are limited, if not unavailable. In this study, we collected whole fish, GI tracts of fish and whole bivalves directly from fish markets or from fisherman selling their catch for human consumption to measure the presence of anthropogenic debris, most specifically plastic debris and fibers from textiles, in seafood. Fish were purchased from the Paotere Fish Market in Makassar, Sulawesi, Indonesia and fish and shellfish were provided by and/or purchased from local fishermen and fish markets in Half Moon Bay and Princeton, California, USA (See Fig. 1 for a map of market locations). Data from both countries were used to help determine if the presence of anthropogenic debris in seafood is widespread and if patterns of occurrence and type of debris may relate to differences in waste management strategies among locations.

## Results

### Anthropogenic Debris in Fish Sampled from Indonesia

From the Paotere Fish Market in Indonesia, we purchased 76 whole fish across 11 different species. The species included 5 tilapia (*Oreochromis niloticus*), 9 skipjack tuna (*Katsuwonus pelamis*), 9 Indian mackerel (*Rastrelliger kanagurta*), 17 shortfin scad (*Decapterus macrosoma*), 10 silver-stripe round herring (*Spratelloides gracilis*), 7 from the family Carangidae (could not be identified to genera), 7 rabbitfish (2 *Siganus argenteus*, 3 *Siganus fuscescens*, 2 *Siganus canaliculatus*), 5 humpback red snapper (*Lutjanus gibbus*) and 7 oxeye scad (*Selar boops*). Overall, 21 out of 76 (28%) fish sampled had anthropogenic debris in their GI tract (Fig. 2). Of the 11 species purchased, anthropogenic debris was present in the gut content of six (55%) species including Indian mackerel, shortfin scad, silver-stripe round herring, fish from the family Carangidae that could not be identified to genera and 2 species of rabbitfish (S. *argenteus and S. canaliculatus*) (Table 1). Within each species of fish, we found anthropogenic debris in 56% of Indian mackerel, 29% of shortfin scad, 40% of herring, 71% from a small or juvenile (~20 cm body length) species of coastal Carangidae and 29% of rabbitfishes. The number of debris particles in individual fish ranged from 0–21 individual pieces (Fig. 2; see Table 1 for ranges in each species and Supplementary Material Table S1 for number of particles in individual fish).

Of the anthropogenic debris identified (>500 μm) in samples from Indonesia, all were composed of plastic. All debris was small: the average length of all anthropogenic debris was 3.5 mm (±1.1 SD) and the width ranged from 0.1–4.5 mm depending on the shape (see Supplementary Material, Table S3 for individual particle sizes of all pieces measured). The 105 total pieces of anthropogenic debris recovered from fish (Fig. 2) included 63 plastic fragments (60%), 0 fibers (0%), 39 pieces of plastic foam (37%), 2 plastic film (2%) and 1 plastic monofilament line (1%; Fig. 3; see Table 1 for types of anthropogenic debris found in each species and Supplementary Material Table S3 for types of anthropogenic debris found in individual fish and Figure S1 for images of several pieces of anthropogenic debris found).

### Anthropogenic Debris in Fish and Shellfish Sampled from the USA

We processed 64 individual fish across 12 different species from California. The fishes included 7 jacksmelt (*Atherinopsis californiensis*), 10 Pacific anchovy (*Engraulis mordax*), 1 Pacific mackerel (*Scomber japonicus*), 3 yellowtail rockfish (*Sebastes flavidus*), 7 striped bass (*Morone saxatilis*), 4 Chinook salmon *(Oncorhynchus tshawytscha*), 2 albacore tuna (*Thunnus alalunga*), 10 blue rockfish (*Sebastes mystinus*), 5 Pacific sanddab (*Citharichthys sordidus*), 11 lingcod (*Ophiodon elongatus*), 1 copper rockfish (*Sebastes caurinus*) and 3 vermilion rockfish (*Sebastes miniatus*). In addition, we processed 12 individual shellfish samples from 1 species, the Pacific oyster (*Crassostrea gigas*). Overall, 16 out of 64 (25%; Fig. 2) individual fish had anthropogenic debris in their GI tract and 4 out of 12 (33%) individual shellfish were contaminated with anthropogenic debris. Of all species purchased, anthropogenic debris was present in the gut content of eight (67%) of all fish species sampled, including jacksmelt, Pacific anchovy, yellowtail rockfish, striped bass, Chinook salmon, blue rockfish, Pacific sanddab and lingcod and in the Pacific oyster (Table 2). Within each species, we found anthropogenic debris in 29% of jacksmelt, 30% of Pacific anchovies, 33% of yellowtail rockfish, 43% of striped bass, 25% of Chinook salmon, 20% of blue rockfish, 60% of Pacific sanddabs, 9% of lingcod and 33% of Pacific oysters. The number of anthropogenic particles in individual fish ranged from 0–10 individual pieces and in individual oysters from 0–2 individual pieces (Fig. 2; see Table 2 for ranges in each species and Supplementary Material Table S2 for number of particles in individual fish).

Of the anthropogenic debris identified (>500 μm) in samples from California, the majority were fibers from textiles. Because we did not have the ability to use FTIR or Raman Spectroscopy to confirm the material type, we cannot be sure if the fibers are made from synthetic material (i.e. plastic) or natural fibers such as cotton. As such, we have categorized the fibers we recovered from fish and shellfish as anthropogenic debris, but not as plastic debris. Only 6 individual fish contained debris that were not fibers and thus could be confidently identified as plastic. There was one plastic fragment in a jacksmelt, one piece of styrofoam in a striped bass, one piece of film each in a Pacific anchovy, a striped bass and a Pacific sanddab and one piece of plastic monofilament in a Pacific anchovy. All debris was small: the average length of all types of anthropogenic debris recovered from fish was 6.3 mm (±6.7 SD) and the width ranged from 0.01–2.1 mm depending on whether it was fibrous or particle-like (see Supplementary Material, Table S4 for individual particle sizes). The average length of all fibers recovered from oysters was 5.5 mm (±5.8 SD) and the width ranged from 0.02–0.05 mm (see Supplementary Material, Table S4 for individual particle sizes). The 30 total pieces of anthropogenic debris recovered from fish (Fig. 2) included 1 fragment (3.33%), 24 fibers (80%), 1 piece of foam (3.33%), 3 film (10%) and 1 monofilament line (3.33%; Fig. 3). All 7 total pieces of anthropogenic debris recovered from Pacific oysters were fibers (100%; see Table 2 for types of anthropogenic debris found in each species, Supplementary Material Table S4 for types of plastic debris found in individual fish and shellfish and Figure S2 for images of several pieces of anthropogenic debris found).

### Differences Among Species

In both locations, anthropogenic debris was found in fishes occupying different trophic levels (herbivores, predators) and habitats (coastal seagrass and reefs, pelagic). Of fish purchased from Indonesia, one species, the tilapia, was from freshwater aquaculture and contained no anthropogenic debris. Four species were pelagic fish (skipjack tuna, Indian mackerel, shortfin scad and silver-stripe round herring)^33^, three of which contained plastic debris. The remaining six species were reef fishes (1 small carangid, 3 species of herbivorous rabbitfishes, red snapper and oxeye scad)^33^, four of which contained plastic debris. Of fish purchased in Indonesia, 67% of individual fishes containing plastic debris represent three of four total species associated with pelagic habitats where they feed on phytoplankton and zooplankton. The other 33% of individuals with plastic were three of six species that reside in reef habitats and feed on seagrass and algae (rabbitfishes) or fish and invertebrates (Carangidae)^33^. Hard fragments and fishing line were found in both pelagic and reef fish, but film and foam were found only in pelagic fish.

Of fish purchased in California, seven species were pelagic (jacksmelt, Chinook salmon, Pacific anchovy, yellowtail rockfish, striped bass, Pacific mackerel and albacore tuna)^33^, five of which contained anthropogenic debris. The remaining five species were demersal (blue rockfish, Pacific sanddab, lingcod copper rockfish and vermilion rockfish)^33^, three of which contained anthropogenic debris. For individual fish, 60% represent five of seven species that reside in pelagic habitats where they feed on phytoplankton, zooplankton and fish. The remaining 40% of individual fish with anthropogenic debris represent three of five species of demersal fishes (i.e., associated with the bottom) and feed on benthic invertebrates and fish^33^. Debris composed of fibers, foam, film, monofilament and a fragment were found in pelagic fish and fibers and film only were found in demersal fish. The Pacific oysters came from aquaculture in urban bays and had anthropogenic debris composed entirely of fibers. These shellfish filter food from the water column, and thus are exposed to urban runoff and wastewater discharge. As such, the presence of fibers, the only type of anthropogenic debris found in Pacific oysters, is not surprising, as reported previously in another study^22^.

### Differences Among Locations

Overall, when considering fish and shellfish, the occurrence of anthropogenic debris in individual animals was slightly greater in Indonesia (28% in Indonesia and 26% in the USA). Similarly, for fish only, anthropogenic debris was found in 28% of fish from Indonesia and 25% in the USA (Fig. 2). For plastic debris only, removing the consideration of fibers because we are unsure whether or not they were made from synthetic polymers, the frequency of occurrence was much greater in Indonesia. In Indonesia plastic debris was found in 28% of fish with confidence, but only in 9% of fish from the USA. The presence of fibers in 0% of fish from Indonesia and in 19% from the USA is worth noting.

Overall, individual animals sampled from Indonesia had greater amounts of individual pieces of anthropogenic debris in their gut content. In total, there were 3× as many individual pieces of debris recovered from animals from Indonesia. 105 pieces of total anthropogenic debris were recovered from 76 individual fish in Indonesia and 30 from 64 individual fish in the USA. For all anthropogenic debris recovered from fish, the average number of pieces per individual fish in Indonesia was 1.4 ± 3.7 SD and in the USA 0.5 ± 1.4 SD. In Indonesia, the number of pieces of anthropogenic debris per individual fish ranged from 0–21, with 8 fish having ≥5 pieces of debris in their gut content. In the USA, the number of pieces of anthropogenic debris per individual fish ranged from 0–10, with only 1 fish having ≥5 pieces of debris in their gut content. Without fibers, in the USA there was only 1 piece of plastic debris in each of six individual fish making the average number of pieces per all individual fish sampled 0.1 ± 0.3 SD (See Supplementary Material Table S2 for number of particles in individual fish). Thus, the discrepancy between Indonesia and the USA is not only due to differences in material type, but also the number of pieces of anthropogenic debris per individual animal.

## Discussion

Our measurements of occurrence and quantity of anthropogenic debris in seafood are conservative, as we did not quantify the particles observed which were smaller than 0.5 mm in every dimension or fibers that matched the color of our lab coats or clothing. Still, across both locations, we found anthropogenic debris in the gut contents of greater than a quarter of individual fishes and shellfish and in the majority of species sampled—all marketed for human consumption. This result may not be surprising given both countries rank among the top 20 for mismanaged anthropogenic waste (Indonesia ranks 2^nd^ and the USA 20^th^)^34^.

Overall, the frequency of occurrence of plastic debris in seafood was similar between locations. We sampled 76 individuals from each location and found anthropogenic debris in 21 from Indonesia and 19 from the USA. For fish only, we found anthropogenic debris in 21 individuals from Indonesia and in 16 from the USA. Although the frequency of debris in fish was similar, there was a trend for individual Indonesian fish to contain a higher number of particles (Fig. 2). This trend may be due to differences in the management of waste between Indonesia and the USA and warrants further analysis. While the use of plastic and textiles in the US is greater than in Indonesia^35^, waste management is more advanced in the US. For example, Indonesia has an order of magnitude greater mismanaged plastic waste than the US (3.22 million metric tons as opposed to 0.28 in the US)^34^.

The most striking difference between locations is the type of anthropogenic debris found in fish between Indonesia and the USA (Fig. 3). All anthropogenic debris found in fish from Indonesia was composed of plastic, whereas in fish from the USA only 20% of anthropogenic debris found in fish could be confirmed as plastic. In contrast, the majority (80%) of anthropogenic debris found in fish from the USA was composed of fibers from textiles, whereas not a single fiber was detected in fish from Indonesia. Many studies report procedural contamination of fibers in samples, and omit fibers from quantification unless laboratory blanks have been used. Here, the same methods were used in Indonesia and the USA, which included laboratory blanks and the exclusion of fibers that matched our laboratory coats and clothing. The lack of fibers in fish from Indonesia helps confirm that our procedures were robust. While there is a chance that gutting of some USA fish by fishermen might have introduced fibers to gut contents, we also found fibers in the guts of whole fish. Thus, we conclude that the presence of fibers in samples from the USA occurred from ingestion in nature prior to sampling.

Although we cannot explain the cause of this stark contrast between types of anthropogenic debris between sampling locations, one hypothesis may concern the differences in waste management strategies on land between countries. In Makassar, Indonesia where fish were collected, 30% of solid waste generated is not processed and an increasing amount of waste is directly discarded along the coast, rivers and into drainage channels^36^; thus, it is common for plastic items to end up in the ocean where they degrade into fragments over time^37^. In California, waste management systems are more advanced and thus it is less common for plastic items to be discarded in the ocean. Together, this may have led to a higher frequency of plastic fragments in fish from Makassar than California. In regards to the contamination from fibers in fish from the USA only, a more advanced waste management system may explain the higher concentration of fibers off the coast of California. There are more than 200 wastewater treatment plants discharging billions of liters of treated final effluent just offshore in California^38^. Even though treatment results in a reduction of many contaminants, synthetic fibers from washing machines can remain in sewage effluent, and may be delivered to aquatic habitats in large concentrations via wastewater outfalls^39^,^40^. One study found one fiber per L of wastewater effluent^39^. In this sense, we might expect that billions of fibers are discarded into the Pacific Ocean from wastewater treatment plants in California everyday. Without this concentrated source of fibers in Makassar, we may expect a lower concentration of fibers in fish from Indonesia. Still, the complete lack of fibers in fish from Indonesia was not expected and future research should test hypotheses about such patterns.

Anthropogenic debris has become widespread in the marine environment globally. As such, concern has been raised about whether the ingestion of anthropogenic debris by marine animals can cascade up the food web to influence human health^1^. There may be direct effects when shellfish, whole fish and/or the intestines of fish are ingested whole. In South Sulawesi fish guts are a local favorite providing a direct route for ingestion of anthropogenic debris by people. The physical harm that anthropogenic debris causes to marine animals at several levels of biological organization^27^ can potentially threaten local food security in locations where debris is abundant and seafood is a major source of protein to the local population (e.g., Indonesian island communities). Moreover, anthropogenic debris is associated with a cocktail of hazardous chemicals^25^, some of which are bioavailable to wildlife upon ingestion. Recent evidence demonstrates that chemicals associated with plastic anthropogenic debris are bioavailable to seabirds^41^,^42^,^43^, amphipods^44^, crickets^45^, lugworms^23^,^46^ and fish^47^ upon ingestion.

Our results, showing anthropogenic debris in more than 25% of individual animals and over half of the species purchased and/or collected from fish markets and fishermen selling fish for human consumption, demonstrate that anthropogenic debris has infiltrated marine foodwebs to the level of humans via seafood. Because anthropogenic debris is associated with a cocktail of priority pollutants^25^,^30^, some of which can transfer to animals upon ingestion^23^,^44^,^45^,^46^,^47^, this work supports concern that chemicals from anthropogenic debris may be transferring to humans via diets containing fish and shellfish, raising important questions regarding the bioaccumulation and biomagnification of chemicals and consequences for human health Our results provide the impetus for further research to test hypotheses about the linkages between plastic contamination of seafood and human health. It is important to understand any hazards associated with how the ghost of waste management past may be haunting us in our own seafood. Future risk assessments should consider anthropogenic debris as another factor for seafood safety advisories relevant for consumers.

## Methods

### Sample Collection

**Figure 1:**
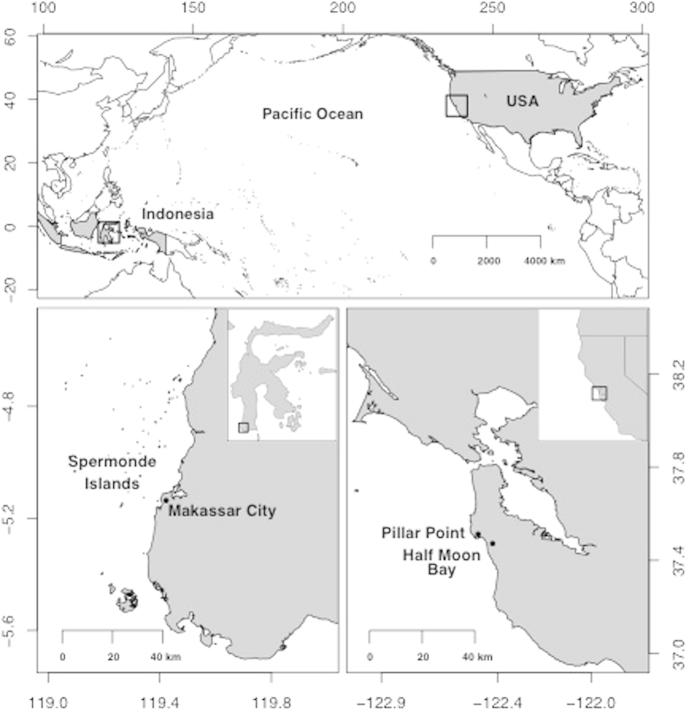
Map of sampling locations. Samples of fish and shellfish (152 total samples) sold for human consumption were collected August through November of 2014 from local fish markets and/or fishermen in Makassar, Sulawesi, Indonesia (bottom left) and Half Moon Bay, California, USA (bottom right). Map produced using the open-source software R library “mapdata”^52^ and Global Administrative Areas (GADM) database^53^.

**Figure 2:**
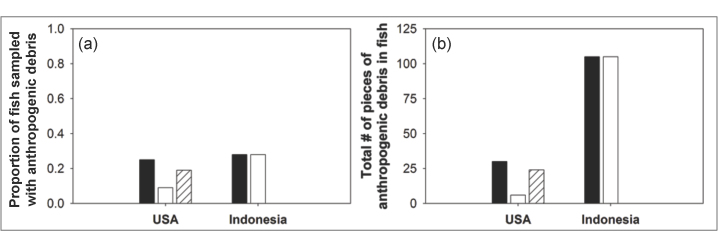
Anthropogenic debris recovered from fish sampled from the USA and Indonesia. The graph on the left (**a**) shows the proportion of individual fish sampled in each location that contained anthropogenic debris (black) in their GI tract. The middle bar (white) shows the proportion with plastic debris and the right bar (diagonal lines) shows the proportion with fibers. The graph on the right (**b**) shows the total number of pieces of anthropogenic debris (black) found across all fish from each location. The middle bar (white) shows the total number of pieces of plastic debris and the right bar (diagonal lines) shows the total number of fibers.

**Figure 3:**
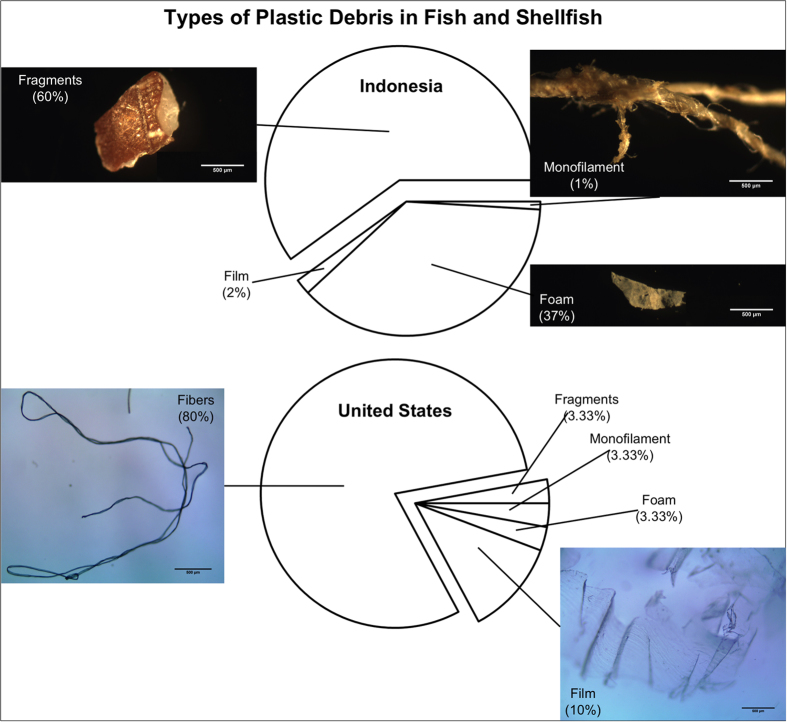
Types of anthropogenic debris in market fish products sampled from Indonesia and the United States. The pie charts above show the percentage of each type (i.e. plastic fragments, fibers, plastic film, plastic foam and plastic monofilament) of anthropogenic debris found across all fish sampled from Indonesia (top) and the United States (bottom). Images show examples of each type of debris found. Scale bars on all pictures are set at 500 μm.

Samples of fish and shellfish (152 total samples) sold for human consumption were collected August through November of 2014 from local fish markets and/or fishermen in Makassar, Sulawesi, Indonesia and Half Moon Bay, California, USA (see Fig. 1 for a map of study locations). We selected fishes that span across habitats (e.g., reef, pelagic, benthic) and trophic levels. In Indonesia, 76 whole fish (2–17 individuals from each of 11 species; see Table 1) were purchased from the Paotere Fish Market on Paotere Harbor in northern Makassar on August 29, 2014. This fish market is the largest of three in the region where the majority of fish are caught in the Spermonde Islands^48^ offshore of the city of Makassar. In California, 76 samples (1–12 individuals from each of 12 species of fish and 1 species of shellfish; see Table 2) were purchased or donated from a local fish market in Half Moon Bay and a local fish market and local fishermen on Pillar Point Harbor in Princeton on October 18^th^ and November 22^nd^ of 2014. Fish and shellfish sold from these markets and fishermen were mostly caught offshore in Central California, and some in Oregon, USA. All shellfish and some species of fish from California were sampled whole, but many fishes were first gutted prior to being sold for human consumption, as is typical in the USA. In these cases, the entire gastrointestinal (GI) tract was donated and the fish market or fishermen provided species identification.

### Analytical Methods

Fish sampled in Indonesia were brought back to the laboratory at the Hasanuddin University and immediately processed for analysis. First, fishes were identified to species where possible and pictures were taken of individual fish for subsequent identification. Fish were then dissected and the GI tract was removed. The GI tract was placed into individual polypropylene sample jars. Following a modified method from Foekema *et al*.^18^, to extract anthropogenic debris from the gut content of fish, each sample jar containing the GI tract was filled to 3× the volume of the tissue with a 10% KOH solution in ultrapure water and incubated overnight at 60 °C to digest organic material. To avoid cross contamination between samples, all tools and glassware were rinsed with ultrapure water three times between samples.

Fish sampled in the USA were brought back to the laboratory at the University of California, Davis on ice and stored at 4 °C until analysis. Following the same standard operating procedure as the dissection of whole fish in Indonesia, fish were cut open and the GI tract was removed. All GI tracts from individual fish and the entire tissue of each shellfish, removed from the shell, were placed into individual glass jars that had been pre-cleaned with ultrapure water and baked at 450 °C for 6 hours. When the GI tract was too large (for example for salmon and albacore tuna), we cut open the GI tract and scraped the contents into each sample jar using a metal spatula. Following the same method above, each sample jar was filled to 3× the volume of the tissue with a 10% KOH solution in ultrapure water and incubated overnight at 60 °C to digest organic material. To avoid cross contamination between samples, all tools and glassware were rinsed with ultrapure water three times between samples.

Anthropogenic debris was extracted and quantified from the gut content of fish or body burden of shellfish using methods employed in previous studies^18^,^44^,^49^,^50^. For each sample, the digested material was sorted and examined carefully under a dissecting microscope using a glass petri dish that had been rinsed with ultrapure water three times. Each piece of anthropogenic debris, larger than 0.5 mm, was recorded and for some individual pieces of debris a photo was taken with a ruler to be used for future measurements. Photographs of debris were used for final confirmation, to identify debris type and to record the size of the material. In Indonesia, the laboratory did not have a camera available to attach to the microscopes and thus pictures for measurements were taken with a cell phone. This limited our ability to take multiple images. Thus, when one animal or several individuals from the same species contained several pieces of debris of a similar type and size a picture of only 1–2 pieces was taken and used for exact measurement. To determine the width and length of each particle of debris, all photographed pieces were digitally measured using the software package ImageJ 1.45^51^.

Our methods included several steps to avoid procedural contamination, cross-contamination and/or misidentification of natural debris (e.g., coral, shells, algae) as anthropogenic. To avoid cross-contamination, all tools and glassware were rinsed three times with ultrapure water between samples. To avoid procedural contamination, we used laboratory blanks composed of cleaned petri dishes filled with ultrapure water in each laboratory. In Indonesia, nothing was found in each of three blanks. In the USA, fibers resembling our laboratory coats were found in two of three laboratory blanks. Thus, any fiber that resembled our lab coats or the color of our clothing on the day of analysis was discarded and not quantified as anthropogenic debris. To avoid misidentification, each piece of anthropogenic debris was confirmed by at least two trained people who had to agree that the material did not resemble something natural. When one person disagreed or was unsure, the material was disregarded. If both people agreed, the debris was measured to assure it was greater than 0.5 mm. Because we could not reliably categorize particles less than 0.5 mm, they were excluded as anthropogenic debris.

## Additional Information

**How to cite this article**: Rochman, C. M. *et al*. Anthropogenic debris in seafood: Plastic debris and fibers from textiles in fish and bivalves sold for human consumption. *Sci. Rep*. **5**, 14340; doi: 10.1038/srep14340 (2015).

## Supplementary Material

Supplementary Information

## Figures and Tables

**Table 1:** Fish purchased from Indonesia.

Common name (*Genus species*)	Number collected	Number with debris	Number of pieces of debris/animal(average (±SD), range)	Types of debris
tilapia (*Oreochromis niloticus*)	5	0	0, 0	N/A
skipjack tuna (*Katsuwonus pelamis*)	9	0	0, 0	N/A
Indian Mackerel (*Rastrelliger kanagurta*)	9	5	1 (±1.1), 0–3	fragment, film, monofilament
shortfin scad (*Decapterus macrosoma*)	17	5	2.5 (±6.3), 0–21	styrofoam, fragments
herring (*Spratelloides gracilis*)	10	4	1.1 (±1.7), 0–5	fragments
Family Carangidae (*??, ??*)	7	5	5.9 (±5.1), 0–14	fragments
rabbitfish (*Siganus argenteus*)	2	1	0.5 (±0.7), 0–1	fragment
rabbitfish (*Siganus fuscescens*)	2	0	0, 0	N/A
rabbitfish (*Siganus canaliculatus*)	3	1	0.3 (±0.6), 0–1	monofilament
humpback red snapper (*Lutjanus gibbus*)	5	0	0, 0	N/A
oxeye scad (*Selar boops*)	7	0	0, 0	N/A

The table shows the common name, genus and species of fish, the number of individual animals purchased, the number of individuals from each group that had anthropogenic debris, the average number of individual pieces of debris in each animal per species group (including individuals where no debris was found), the range of individual pieces of debris per animal in each group and the types of debris found in each group.

**Table 2:** Fish and shellfish purchased from the USA.

Common name (*Genus species*)	Number collected	Number with debris	Number of pieces of debris/animal (average (±SD), range)	Types of debris
Pacific oyster (*Crassostrea gigas*)	12	4	0.6 (±0.9), 0–2	fibers
jacksmelt (*Atherinopsis californiensis*)	7	2	1.6 (±3.7), 0–10	fibers, fragment
Pacific anchovy (*Engraulis mordax*)	10	3	0.3 (±0.5), 0–1	fiber, film, monofilament
Pacific mackerel (*Scomber japonicus*)	1	0	0, 0	N/A
yellowtail Rockfish (*Sebastes flavidus*)	3	1	0.3 (±0.6), 0–1	fiber
striped bass (*Morone saxatilis*)	7	2	0.9 (±1.2), 0–3	fiber, film, foam
Chinook salmon *(Oncorhynchus tshawytscha*)	4	1	0.25 (±0.5), 0–1	fiber
albacore tuna (*Thunnus alalunga*)	2	0	0, 0	N/A
blue rockfish (*Sebastes mystinus*)	10	2	0.2 (±0.4), 0–1	fibers
Pacific sanddab (*Citharichthys sordidus*)	5	3	1 (±1.2), 0–3	fiber, film
lingcod (*Ophiodon elongatus*)	11	1	0.1(±0.3), 0–1	film
copper rockfish (*Sebastes caurinus*)	1	0	0, 0	N/A
vermilion rockfish (*Sebastes miniatus*)	3	0	0, 0	N/A

The table shows the common name, genus and species of fish or shellfish, the number of individual animals purchased, the number of individuals from each group that had anthropogenic debris, the average number of individual pieces of debris in each animal per species group (including individuals where no debris was found), the range of individual pieces of debris per animal in each group and the types of debris found in each group.
