# Supplementary material for: Anthropogenic debris in seafood: Plastic debris and fibers from textiles in fish and bivalves sold for human consumption
Source: Sci Rep. 2015 Sep 24;5:14340. doi: 10.1038/srep14340 (PMC4585829; doi:10.1038/srep14340)
Supplement: Supplementary Information [file srep14340-s1.pdf]

Supplementary Material

Anthropogenic debris in seafood: Plastic debris and fibers from textiles in fish and bivalves sold for human consumption

Rochman, Chelsea M[1*]; Tahir, Akbar[2]; Williams, Susan L.[3]; Baxa, Dolores V[1]; Lam, Rosalyn[1]; Miller, Jeffrey T[4]; Teh, Foo-Ching[1]; Werorilangi, Shinta[2]; Teh, Swee J[1]

[1]Aquatic Health Program, School of Veterinary Medicine, University of California, Davis, Davis, CA 95616, USA.

[2]Department of Marine Science, Faculty Marine and Fisheries Sciences, University of Hasanuddin, Makassar 90245, Indonesia.

[3] Bodega Marine Laboratory and Department of Evolution and Ecology, University of California at Davis, Bodega Bay, CA 94923, USA

[4]Department of Environmental Toxicology, University of California, Davis, Davis, CA, 95616, USA

Table of Contents

Table S1. Data from each individual fish sampled from Indonesia

Table S2. Data from each individual fish sampled from USA

Table S3. Type and size of anthropogenic debris sampled from Indonesia

Table S4. Type and size of anthropogenic debris sampled from the USA

Figure S1. Images of individual pieces of anthropogenic debris found in samples from Indonesia.

Figure S2. Images of individual pieces of anthropogenic debris found in samples from California.

Table S1. Data from each individual fish sampled from Indonesia

Sample #	Location	common name (*Genus, species*)	No. Pieces of Anthropogenic Debris	No. Pieces of Plastic Debris	No. of Fibers
1	Makassar, Indonesia	tilapia (*Oreochromis niloticus*)	0	0	0
2	Makassar, Indonesia	tilapia (*Oreochromis niloticus*)	0	0	0
3	Makassar, Indonesia	tilapia (*Oreochromis niloticus*)	0	0	0
4	Makassar, Indonesia	tilapia (*Oreochromis niloticus*)	0	0	0
5	Makassar, Indonesia	tilapia (*Oreochromis niloticus*)	0	0	0
6	Makassar, Indonesia	skipjack tuna (*Katsuwonus pelamis*)	0	0	0
7	Makassar, Indonesia	skipjack tuna (*Katsuwonus pelamis*)	0	0	0
8	Makassar, Indonesia	skipjack tuna (*Katsuwonus pelamis*)	0	0	0
9	Makassar, Indonesia	skipjack tuna (*Katsuwonus pelamis*)	0	0	0
10	Makassar, Indonesia	skipjack tuna (*Katsuwonus pelamis*)	0	0	0
11	Makassar, Indonesia	skipjack tuna (*Katsuwonus pelamis*)	0	0	0
12	Makassar, Indonesia	skipjack tuna (*Katsuwonus pelamis*)	0	0	0
13	Makassar, Indonesia	skipjack tuna (*Katsuwonus pelamis*)	0	0	0
14	Makassar, Indonesia	skipjack tuna (*Katsuwonus pelamis*)	0	0	0
15	Makassar, Indonesia	indian Mackerel (*Rastrelliger kanagurta*)	2	2	0
16	Makassar, Indonesia	indian Mackerel (*Rastrelliger kanagurta*)	0	0	0
17	Makassar, Indonesia	indian Mackerel (*Rastrelliger kanagurta*)	1	1	0
18	Makassar, Indonesia	indian Mackerel (*Rastrelliger kanagurta*)	1	1	0
19	Makassar, Indonesia	indian Mackerel (*Rastrelliger kanagurta*)	0	0	0
20	Makassar, Indonesia	indian Mackerel (*Rastrelliger kanagurta*)	2	2	0
21	Makassar, Indonesia	indian Mackerel (*Rastrelliger kanagurta*)	3	3	0
22	Makassar, Indonesia	indian Mackerel (*Rastrelliger kanagurta*)	0	0	0
23	Makassar, Indonesia	indian Mackerel (*Rastrelliger kanagurta*)	0	0	0
24	Makassar, Indonesia	shortfin scad (*Decapterus macrosoma*)	0	0	0
25	Makassar, Indonesia	shortfin scad (*Decapterus macrosoma*)	2	2	0
26	Makassar, Indonesia	shortfin scad (*Decapterus macrosoma*)	17	17	0
27	Makassar, Indonesia	shortfin scad (*Decapterus macrosoma*)	21	21	0
28	Makassar, Indonesia	shortfin scad (*Decapterus macrosoma*)	0	0	0
29	Makassar, Indonesia	shortfin scad (*Decapterus macrosoma*)	1	1	0
30	Makassar, Indonesia	shortfin scad (*Decapterus macrosoma*)	1	1	0
31	Makassar, Indonesia	shortfin scad (*Decapterus macrosoma*)	0	0	0
32	Makassar, Indonesia	shortfin scad (*Decapterus macrosoma*)	0	0	0
33	Makassar, Indonesia	shortfin scad (*Decapterus macrosoma*)	0	0	0
34	Makassar, Indonesia	shortfin scad (*Decapterus macrosoma*)	0	0	0
35	Makassar, Indonesia	shortfin scad (*Decapterus macrosoma*)	0	0	0
36	Makassar, Indonesia	shortfin scad (*Decapterus macrosoma*)	0	0	0
37	Makassar, Indonesia	shortfin scad (*Decapterus macrosoma*)	0	0	0
38	Makassar, Indonesia	shortfin scad (*Decapterus macrosoma*)	0	0	0
39	Makassar, Indonesia	shortfin scad (*Decapterus macrosoma*)	0	0	0
40	Makassar, Indonesia	shortfin scad (*Decapterus macrosoma*)	0	0	0
41	Makassar, Indonesia	herring (*Spratelloides gracilis*)	0	0	0
42	Makassar, Indonesia	herring (*Spratelloides gracilis*)	5	5	0
43	Makassar, Indonesia	herring (*Spratelloides gracilis*)	1	1	0
44	Makassar, Indonesia	herring (*Spratelloides gracilis*)	0	0	0
45	Makassar, Indonesia	herring (*Spratelloides gracilis*)	0	0	0
46	Makassar, Indonesia	herring (*Spratelloides gracilis*)	3	3	0
47	Makassar, Indonesia	herring (*Spratelloides gracilis*)	0	0	0
48	Makassar, Indonesia	herring (*Spratelloides gracilis*)	0	0	0
49	Makassar, Indonesia	herring (*Spratelloides gracilis*)	2	2	0
50	Makassar, Indonesia	herring (*Spratelloides gracilis*)	0	0	0
51	Makassar, Indonesia	family Carangidae (*??, ??*)	10	10	0
52	Makassar, Indonesia	family Carangidae (*??, ??*)	5	5	0
53	Makassar, Indonesia	family Carangidae (*??, ??*)	0	0	0
54	Makassar, Indonesia	family Carangidae (*??, ??*)	14	14	0
55	Makassar, Indonesia	family Carangidae (*??, ??*)	5	5	0
56	Makassar, Indonesia	family Carangidae (*??, ??*)	0	0	0
57	Makassar, Indonesia	family Carangidae (*??, ??*)	7	7	0
58	Makassar, Indonesia	rabbitfish (*Siganus argenteus*)	1	1	0
59	Makassar, Indonesia	rabbitfish (*Siganus argenteus*)	0	0	0
60	Makassar, Indonesia	rabbitfish (*Siganus fuscescens*)	0	0	0
61	Makassar, Indonesia	rabbitfish (*Siganus fuscescens*)	0	0	0
62	Makassar, Indonesia	rabbitfish (*Siganus canaliculatus*)	0	0	0
63	Makassar, Indonesia	rabbitfish (*Siganus canaliculatus*)	1	1	0
64	Makassar, Indonesia	rabbitfish (*Siganus canaliculatus*)	0	0	0
65	Makassar, Indonesia	humpback red snapper (*Lutjanus gibbus*)	0	0	0
66	Makassar, Indonesia	humpback red snapper (*Lutjanus gibbus*)	0	0	0
67	Makassar, Indonesia	humpback red snapper (*Lutjanus gibbus*)	0	0	0
68	Makassar, Indonesia	humpback red snapper (*Lutjanus gibbus*)	0	0	0
69	Makassar, Indonesia	humpback red snapper (*Lutjanus gibbus*)	0	0	0
70	Makassar, Indonesia	oxeye scad (*Selar boops*)	0	0	0
71	Makassar, Indonesia	oxeye scad (*Selar boops*)	0	0	0
72	Makassar, Indonesia	oxeye scad (*Selar boops*)	0	0	0
73	Makassar, Indonesia	oxeye scad (*Selar boops*)	0	0	0
74	Makassar, Indonesia	oxeye scad (*Selar boops*)	0	0	0
75	Makassar, Indonesia	oxeye scad (*Selar boops*)	0	0	0
76	Makassar, Indonesia	oxeye scad (*Selar boops*)	0	0	0

Table S2. Data from each individual fish and bivalve sampled from USA

Sample #	Location	common name (*Genus, species*)	No. Pieces of Anthropogenic Debris	No. Pieces of Plastic Debris	No. of Fibers
1	California, USA	pacific oyster (*Crassostrea gigas*)	0	0	0
2	California, USA	pacific oyster (*Crassostrea gigas*)	2	0	2
3	California, USA	pacific oyster (*Crassostrea gigas*)	0	0	0
4	California, USA	pacific oyster (*Crassostrea gigas*)	0	0	0
5	California, USA	pacific oyster (*Crassostrea gigas*)	2	0	2
6	California, USA	pacific oyster (*Crassostrea gigas*)	0	0	0
7	California, USA	pacific oyster (*Crassostrea gigas*)	0	0	0
8	California, USA	pacific oyster (*Crassostrea gigas*)	0	0	0
9	California, USA	pacific oyster (*Crassostrea gigas*)	1	0	1
10	California, USA	pacific oyster (*Crassostrea gigas*)	0	0	0
11	California, USA	pacific oyster (*Crassostrea gigas*)	0	0	0
12	California, USA	pacific oyster (*Crassostrea gigas*)	2	0	2
13	California, USA	jacksmelt (*Atherinopsis californiensis*)	0	0	0
14	California, USA	jacksmelt (*Atherinopsis californiensis*)	0	0	0
15	California, USA	jacksmelt (*Atherinopsis californiensis*)	0	0	0
16	California, USA	jacksmelt (*Atherinopsis californiensis*)	1	1	0
17	California, USA	jacksmelt (*Atherinopsis californiensis*)	0	0	0
18	California, USA	jacksmelt (*Atherinopsis californiensis*)	10	0	10
19	California, USA	jacksmelt (*Atherinopsis californiensis*)	0	0	0
20	California, USA	pacific anchovy (*Engraulis mordax*)	0	0	0
21	California, USA	pacific anchovy (*Engraulis mordax*)	0	0	0
22	California, USA	pacific anchovy (*Engraulis mordax*)	0	0	0
23	California, USA	pacific anchovy (*Engraulis mordax*)	1	1	0
24	California, USA	pacific anchovy (*Engraulis mordax*)	0	0	0
25	California, USA	pacific anchovy (*Engraulis mordax*)	1	0	1
26	California, USA	pacific anchovy (*Engraulis mordax*)	0	0	0
27	California, USA	pacific anchovy (*Engraulis mordax*)	0	0	0
28	California, USA	pacific anchovy (*Engraulis mordax*)	1	1	0
29	California, USA	pacific anchovy (*Engraulis mordax*)	0	0	0
30	California, USA	pacific mackerel (*Scomber japonicus*)	0	0	0
31	California, USA	yellowtail rockfish (*Sebastes flavidus*)	0	0	0
32	California, USA	yellowtail rockfish (*Sebastes flavidus*)	1	0	1
33	California, USA	yellowtail rockfish (*Sebastes flavidus*)	0	0	0
34	California, USA	striped bass (*Morone saxatilis*)	2	1	1
35	California, USA	striped bass (*Morone saxatilis*)	3	1	2
36	California, USA	striped bass (*Morone saxatilis*)	1	0	1
37	California, USA	striped bass (*Morone saxatilis*)	0	0	0
38	California, USA	striped bass (*Morone saxatilis*)	0	0	0
39	California, USA	striped bass (*Morone saxatilis*)	0	0	0
40	California, USA	striped bass (*Morone saxatilis*)	0	0	0
41	California, USA	chinook salmon (*Oncorhynchus tshawytscha*)	0	0	0
42	California, USA	chinook salmon (*Oncorhynchus tshawytscha*)	0	0	0
43	California, USA	chinook salmon (*Oncorhynchus tshawytscha*)	1	0	1
44	California, USA	chinook salmon (*Oncorhynchus tshawytscha*)	0	0	0
45	California, USA	albacore tuna (*Thunnus alalunga*)	0	0	0
46	California, USA	albacore tuna (*Thunnus alalunga*)	0	0	0
47	California, USA	blue rockfish (*Sebastes mystinus*)	0	0	0
48	California, USA	blue rockfish (*Sebastes mystinus*)	1	0	1
49	California, USA	blue rockfish (*Sebastes mystinus*)	0	0	0
50	California, USA	blue rockfish (*Sebastes mystinus*)	0	0	0
51	California, USA	blue rockfish (*Sebastes mystinus*)	0	0	0
52	California, USA	blue rockfish (*Sebastes mystinus*)	0	0	0
53	California, USA	blue rockfish (*Sebastes mystinus*)	0	0	0
54	California, USA	blue rockfish (*Sebastes mystinus*)	0	0	0
55	California, USA	blue rockfish (*Sebastes mystinus*)	0	0	0
56	California, USA	blue rockfish (*Sebastes mystinus*)	1	1	1
57	California, USA	pacific sanddab (*Citharichthys sordidus*)	1	0	1
58	California, USA	pacific sanddab (*Citharichthys sordidus*)	1	0	1
59	California, USA	pacific sanddab (*Citharichthys sordidus*)	3	1	2
60	California, USA	pacific sanddab (*Citharichthys sordidus*)	0	0	0
61	California, USA	pacific sanddab (*Citharichthys sordidus*)	0	0	0
62	California, USA	lingcod (*Ophiodon elongatus*)	1	0	1
63	California, USA	lingcod (*Ophiodon elongatus*)	0	0	0
64	California, USA	lingcod (*Ophiodon elongatus*)	0	0	0
65	California, USA	lingcod (*Ophiodon elongatus*)	0	0	0
66	California, USA	lingcod (*Ophiodon elongatus*)	0	0	0
67	California, USA	lingcod (*Ophiodon elongatus*)	0	0	0
68	California, USA	lingcod (*Ophiodon elongatus*)	0	0	0
69	California, USA	lingcod (*Ophiodon elongatus*)	0	0	0
70	California, USA	lingcod (*Ophiodon elongatus*)	0	0	0
71	California, USA	lingcod (*Ophiodon elongatus*)	0	0	0
72	California, USA	lingcod (*Ophiodon elongatus*)	0	0	0
73	California, USA	copper rockfish (*Sebastes caurinus*)	0	0	0
74	California, USA	vermilion rockfish (*Sebastes miniatus*)	0	0	0
75	California, USA	vermilion rockfish (*Sebastes miniatus*)	0	0	0
76	California, USA	vermilion rockfish (*Sebastes miniatus*)	0	0	0

Table S3. Type and size of each piece of anthropogenic debris sampled from Indonesia. N/A denotes where a picture was not available or acceptable to measure the size of the debris.

Sample #	Debris Type	length (mm)	width (mm)
15	plastic fragment	1.3	1
15	plastic fragment	1.4	0.7
17	plastic fragment	4.9	0.8
18	plastic fragment	N/A	N/A
20	plastic fragment	2.4	1.3
20	plastic fragment	2.2	0.8
21	plastic film	N/A	N/A
21	plastic fragment	N/A	N/A
21	plastic fragment	N/A	N/A
25	plastic fragment	N/A	N/A
25	plastic fragment	N/A	N/A
26	plastic foam	0.6	1.1
26	plastic foam	N/A	N/A
26	plastic foam	N/A	N/A
26	plastic foam	N/A	N/A
26	plastic foam	N/A	N/A
26	plastic foam	N/A	N/A
26	plastic foam	N/A	N/A
26	plastic foam	N/A	N/A
26	plastic foam	N/A	N/A
26	plastic foam	N/A	N/A
26	plastic foam	N/A	N/A
26	plastic foam	N/A	N/A
26	plastic foam	N/A	N/A
26	plastic foam	N/A	N/A
26	plastic foam	N/A	N/A
26	plastic foam	N/A	N/A
26	plastic foam	N/A	N/A
27	plastic foam	0.5	0.4
27	plastic foam	N/A	N/A
27	plastic foam	N/A	N/A
27	plastic foam	N/A	N/A
27	plastic foam	N/A	N/A
27	plastic foam	N/A	N/A
27	plastic foam	N/A	N/A
27	plastic foam	N/A	N/A
27	plastic foam	N/A	N/A
27	plastic foam	N/A	N/A
27	plastic foam	N/A	N/A
27	plastic foam	N/A	N/A
27	plastic foam	N/A	N/A
27	plastic foam	N/A	N/A
27	plastic foam	N/A	N/A
27	plastic foam	N/A	N/A
27	plastic foam	N/A	N/A
27	plastic foam	N/A	N/A
27	plastic foam	N/A	N/A
27	plastic foam	N/A	N/A
27	plastic foam	N/A	N/A
29	plastic fragment	N/A	N/A
30	plastic foam	N/A	N/A
42	plastic film	0.6	0.7
42	plastic fragment	N/A	N/A
42	plastic fragment	N/A	N/A
42	plastic fragment	N/A	N/A
42	plastic fragment	N/A	N/A
43	plastic fragment	0.7	0.4
46	plastic fragment	N/A	N/A
46	plastic fragment	2.3	1.6
46	plastic fragment	N/A	N/A
49	plastic fragment	1.2	0.8
49	plastic fragment	0.9	4.5
51	plastic fragment	N/A	N/A
51	plastic fragment	N/A	N/A
51	plastic fragment	N/A	N/A
51	plastic fragment	N/A	N/A
51	plastic fragment	N/A	N/A
51	plastic fragment	N/A	N/A
51	plastic fragment	N/A	N/A
51	plastic fragment	N/A	N/A
51	plastic fragment	N/A	N/A
51	plastic fragment	0.6	0.8
52	plastic fragment	0.8	0.6
52	plastic fragment	N/A	N/A

Sample #	Debris Type	length (mm)	width (mm)
52	plastic fragment	N/A	N/A
52	plastic fragment	N/A	N/A
52	plastic fragment	N/A	N/A
54	plastic fragment	N/A	N/A
54	plastic fragment	N/A	N/A
54	plastic fragment	N/A	N/A
54	plastic fragment	N/A	N/A
54	plastic fragment	N/A	N/A
54	plastic fragment	N/A	N/A
54	plastic fragment	N/A	N/A
54	plastic fragment	N/A	N/A
54	plastic fragment	N/A	N/A
54	plastic fragment	N/A	N/A
54	plastic fragment	N/A	N/A
54	plastic fragment	N/A	N/A
54	plastic fragment	N/A	N/A
54	plastic fragment	N/A	N/A
55	plastic fragment	N/A	N/A
55	plastic fragment	N/A	N/A
55	plastic fragment	N/A	N/A
55	plastic fragment	N/A	N/A
55	plastic fragment	N/A	N/A
57	plastic fragment	N/A	N/A
57	plastic fragment	N/A	N/A
57	plastic fragment	N/A	N/A
57	plastic fragment	N/A	N/A
57	plastic fragment	N/A	N/A
57	plastic fragment	N/A	N/A
57	plastic fragment	N/A	N/A
58	plastic fragment	2	3.1
63	plastic monofilament	36.56	0.198

Table S4. Type and size of each piece of anthropogenic debris sampled from the USA. N/A denotes where a picture was not available or acceptable to measure the size of the debris.

Sample #	Debris Type	length (mm)	width (mm)
2	fiber	N/A	N/A
2	fiber	N/A	N/A
5	fiber	3.281	0.049
5	fiber	15.841	0.02
9	fiber	2.273	0.04
12	fiber	3.521	0.029
12	fiber	2.451	0.036
16	plastic fragment	2.593	0.438
18	fiber	2.68	0.02
18	fiber	4.792	0.024
18	fiber	2.252	0.019
18	fiber	3.322	0.019
18	fiber	N/A	N/A
18	fiber	N/A	N/A
18	fiber	N/A	N/A
18	fiber	N/A	N/A
18	fiber	N/A	N/A
18	fiber	N/A	N/A
23	film	1.115	1.005
25	fiber	6.298	0.029
28	plastic monofilament	16.872	0.022
32	fiber	3.445	0.024
34	fiber	7.424	0.031
34	film	19.985	2.07
35	fiber	6.077	0.021
35	fiber	3.405	0.029
35	foam	0.375	0.563
36	fiber	6.448	0.026
43	fiber	28.8	0.036
48	fiber	2.4	0.016
56	fiber	N/A	N/A
57	fiber	2.688	0.008
58	fiber	4.959	0.021
59	fiber	5.269	0.014
59	fiber	6.155	0.033
59	film	3.312	1.314
62	fiber	3.445	0.024

Figure S1. Images of several of the individual pieces of anthropogenic debris found in samples from Indonesia.

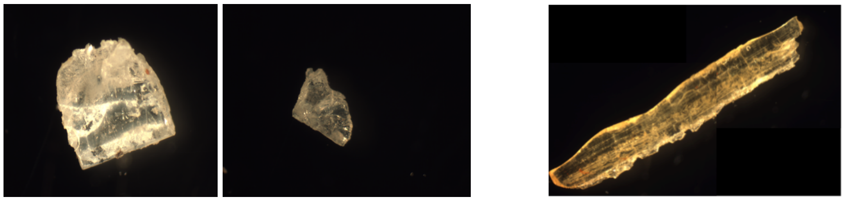

Sample 15 Sample 17

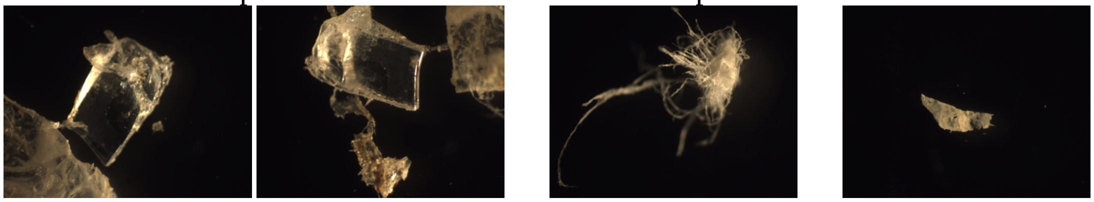

Sample 20 Sample 21 Sample 26

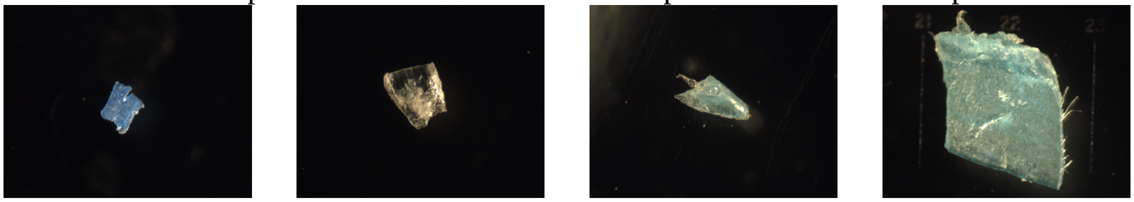

Sample 27 Sample 42 Sample 43 Sample 46

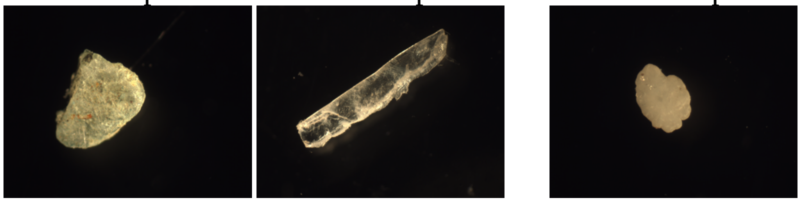

Sample 49 Sample 51

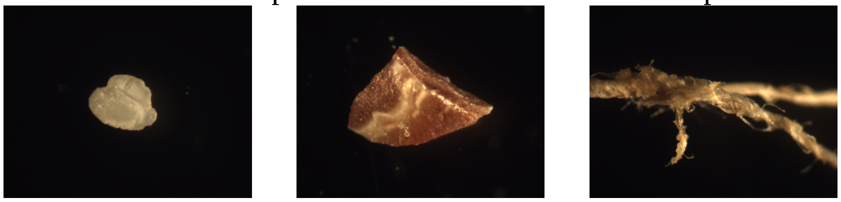

Sample 52 Sample 58 Sample 63

Figure S2. Images of several of the individual pieces of anthropogenic debris found in samples from California.

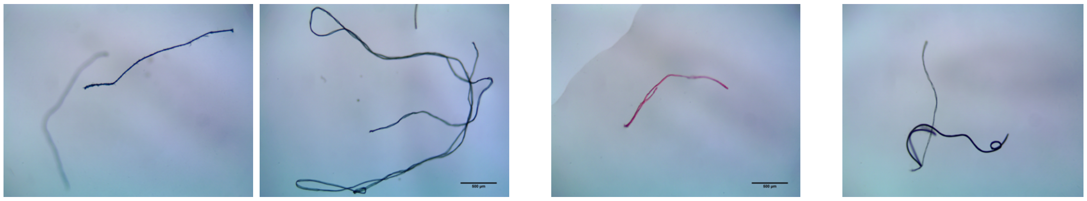

Sample 5 Sample 9 Sample 12 (2 fibers)

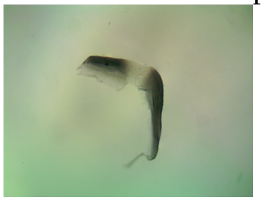

Sample 16

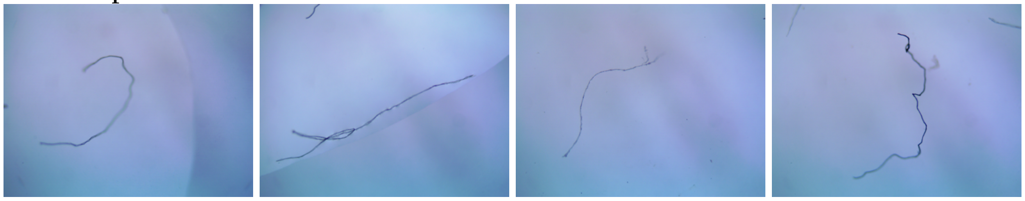

Sample 18

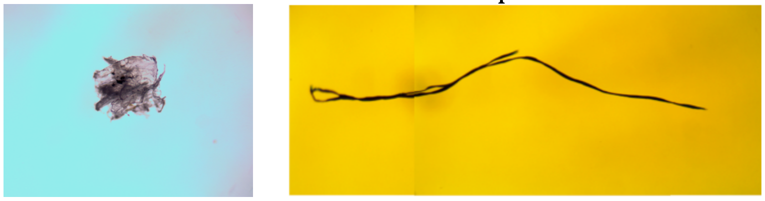

Sample 23 Sample 25

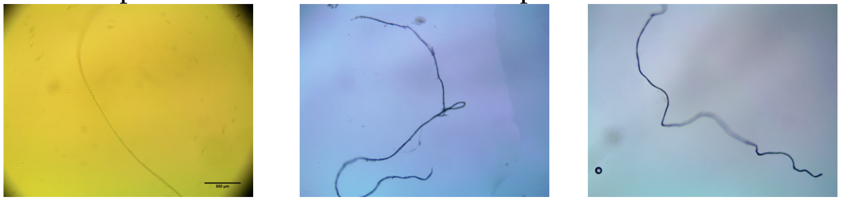

Sample 28 Sample 32 Sample 34

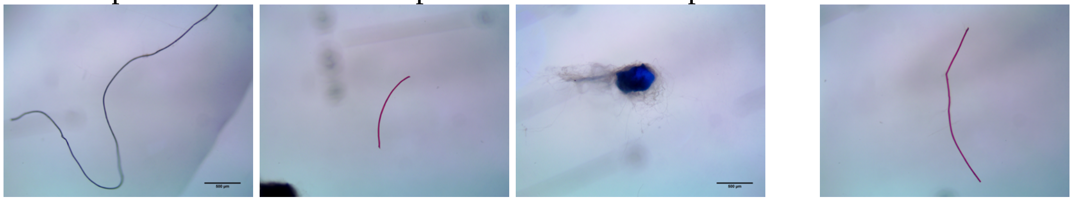

Sample 35 Sample 36

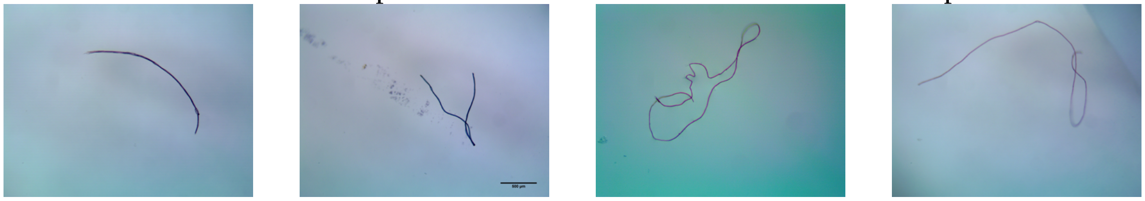

Sample 43 Sample 48 Sample 57 Sample 58

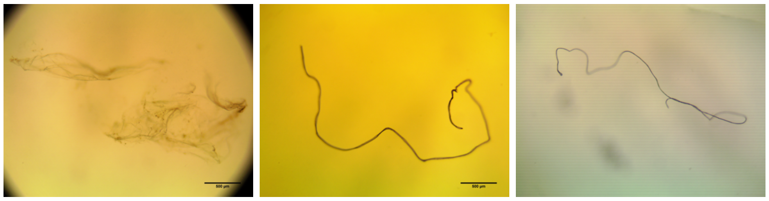

Sample 59

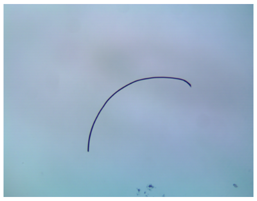

Sample 62
